# Investigation of antimicrobial effects of treated *Lucilia sericata* larvae extract on bacteria

**Published:** 2018-12

**Authors:** Maryam Kaihanfar, Madjid Momeni-Moghaddam, Mohammad Javad Mehdipour Moghaddam, Toktam Hajar, Vahab Dast Pak, Jalal Omrani Bidi

**Affiliations:** 1 Department of Biology, Faculty of Sciences, Hakim Sabzevari University, Sabzevar, Iran; 2 Department of Biology, Faculty of Sciences, University of Guilan, Rasht, Iran; 3 Pharmaceutical Technology Development Center, Jundishapur University of Medical Sciences, Ahvaz, Iran

**Keywords:** Larval therapy, *Luciliasericata*, Wound healing, Bacterial infection

## Abstract

**Background and Objectives:**

Larval therapy refers to the use of *Lucilia sericata* larvae on chronic wounds, which is a successful method of chronic wounds treatment. The secretions of these larvae contain antibacterial compounds and lead to death or inhibition of bacterial growth.

**Materials and Methods:**

In this study, we investigated the antibacterial effects of *Lucilia sericata* larvae secretions which were in sterilized and multi antibiotic-resistant bacteria-treated forms on Gram-positive *Bacillus subtilis* bacteria and Gram-negative *Escherichia coli* bacteria. In the following, we evaluated changes in gene expression of lucifensin and attacin during treatment with multi antibiotic-resistant bacteria. Investigation of the antibacterial effect was carried out using optical absorption and antibiotic disk diffusion in order to study the expression of the aforementioned genes.

**Results:**

The results of this study showed that *E. coli*-treated larvae were able to inhibit the growth of *E. coli* and secretions of *B. subtilis*-treated larvae and were also able to inhibit the growth of *B. subtilis*. Gene expression of antibacterial peptides in multi antibiotic-resistant bacteria-treated larvae was increased in comparison to non-treated larvae.

**Conclusion:**

Due to the significant antibacterial potency of bacteria-treated larvae secretions, the secretions can be a suitable candidate as a drug against antibiotic resistant bacteria, but additional tests are required. Since the antimicrobial peptides of insects have not yet produced any resistance in human pathogenic bacteria, they can be considered as a promising strategy for dealing with resistant infections.

## INTRODUCTION

Skin acts as a physical protection against environmental damage, especially infections, and help maintain the body homeostasis. Any disruption in the integrity of the skin structure can lead to pathological infections or loss of body fluids (1). Therefore, recovery of skin damage will be an important factor in maintaining a healthy body. Sometimes acute wounds do not follow a regular pattern of recovery and become a chronic wound. Chronic wound is a wound that has stopped at one stage of recovery. In most cases, wound healing fails to fight against drug-resistant bacteria and is stopped at the inflammation stage (2). Inappropriate use of antibacterial drugs is the most important reason in spreading multidrug resistant bacteria (3). The need to find a new therapeutic approach is highly vital due to the increasing incurable wounds and increasing antibiotics-resistant bacteria. Larvae therapy is a successful method for eliminating bacteria present in chronic wounds (4).

Larvae therapy is a restorative method in which sterile and live larvae are placed on the wound and these larvae carry out wound recovery (5). Larvae of *Lucilia sericata* fly is the most commonly used species in maggot therapy because of nutrition from necrotic tissue and unwillingness to leave the tissue. It is believed that placement of the fly larvae in an abacterial-contaminated environment (such as a wound area) leads to production of antimicrobial factors in the larvae body and secretion of these factors to the exterior as well (6).

Antimicrobial peptides are always an important part of an insect's immune system against bacterial contaminations (7). Lucifencin is the most important antibacterial peptide in larva secretions (4). Lucifencin is an antibacterial peptide belonging to the defensing family (8). Insect's defensing are medium sized cationic peptide fragments containing di-sulfide bridges. The antibacterial activity of these peptide fragments penetrate into the cell membrane of bacteria and create a hole in the membrane, which leads to loss of potassium and membrane depolarization (7).

Since the lucifensin is detected both inside and outside of the larvae, it can be said that Lucifensin is produced in the body of the larvae and then is secreted out of the body (9). Attacin is a peptide that has an effect on the structure of the outer membrane of the bacteria and inhibits the production of the membrane proteins (11). Identifying active components in antibacterial function will help in finding and designing new therapeutic approachs in the future. In the present study, the antibacterial effect of *Lucilia sericata* larvae's secretions was compared to both growths of Gram-positive and Gram-negative bacteria as well as the placement effect of these larvae in the multi antibiotic resistant bacteria-contaminated environment. In addition, alteration in gene expression of antibacterial compounds was investigated.

## MATERIALS AND METHODS

### Breeding and feeding larvae.

In order to simulate the infectious area of the wound, the larvae were infected through feeding with an infected meat. From stoke of each *B. subtilis* and *E. coli* bacteria (All were multi antibiotics-resistant bacterial strains and were taken from chronic wounds), 10 μl was taken and poured in 1 ml of LB liquid culture medium and it was placed for 12 hours at 37°C and aeration was carried out at 180 rpm. Two pieces of fresh beef, weighing two grams, was prepared and each piece of meat was contaminated with 200 μl of each bacterial strains.

500 sterilized *Lucilia sericata* larvae were received. 2 gr of *B. subtilis* and *E. coli*-contaminated meat were placed into Falcons 1 and 2, respectively as well as 2 grams of sterile meat was placed into Falcon 3. Falcons were placed in incubators at 25°C. Five hours later, in the same way, larvae were fed again. Falcons containing larvae were transferred back to the incubator at 25°C. At this stage, 3 grams of meat were used for feeding and the volume of the bacterial culture medium for infecting meats was increased equally as well. Then, the larvae were incubated at 25°C.

### Extraction of larval secretions.

Four hours after the last feeding and before the larvae entered into the pupa period, the larvae were removed from the incubator and they were washed out with autoclaved distilled water. From each treatment, 160 larvae were taken and larvae of each treatment mixed with 50 ml of distilled water and were placed in a glass plate (3 glass plates andeach plate contained 160 larvae in 50 ml distilled water) and were placed in an incubator at 25°C. Larval secretions were passed through a 0.22 μm filter and were kept at −70°C.

### Studies Molecular.

The GAPDH gene was selected as the reference gene. Forward and reverse primers of GAPDH, Lucifesin and Attacin were designed based on mRNA sequence with annealing temperature of 58–62. The primers are listed below:

Forward GAPH: 5/-ACATCAACTGGGCTAG-CG-3/,
Reverse GAPH: TGAGACCTTCAACGATTTC-CC-3/
Forward Lucifensin : 5/ TCTGCTTGGCTTT-GAGCTTT-3/
Reverse Lucifencin: 5/ ACAATAACCGCCAC-GATTTC-3/
Forward attacin: 5/TGGTACTCCCGAACA-CAATC-3/
Reverse attacin: 5/ ACCATGACCATTAC-GTTCG-3/

Total RNA was extracted using Trypure (Bioneer). The volume of total RNA extracted from each treatment with a concentration of 1μg was used to synthesize cDNA (Using the Bioneer ready to use kit). Real-Time PCR was used to measure changes in gene expression.

## RESULTS

### Studies of antimicrobial effects.

For each bacterial strain which was in liquid culture medium, diagram of optical absorption changes at wavelength of 600 nm was drawn. The rate of the optical absorption had direct correlation with the number of bacterial numbers.

**Fig. 1.**
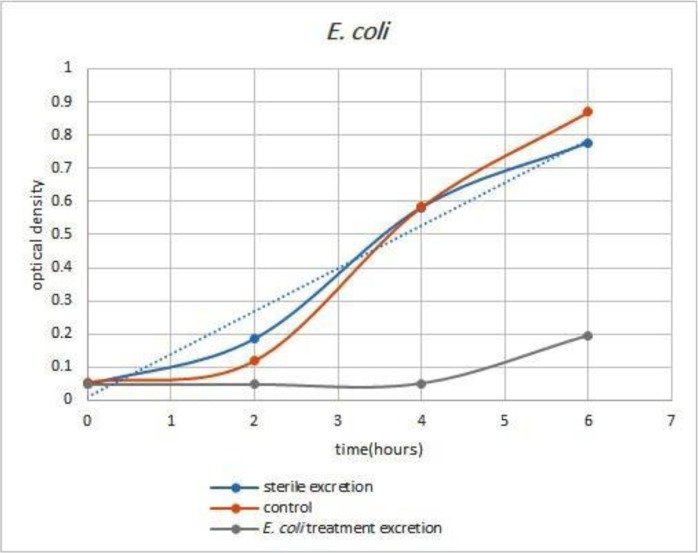
**Rate of bacterial growth.** Gray graph: growth of *E. coli* in vicinity of secretions of *E. coli* bacteria-treated larvae. Blue graph: growth of *E. coli* bacteria in vicinity of secretions of sterile larvae. Red graph: growth of *E. coli* bacteria as control.

**Fig. 2.**
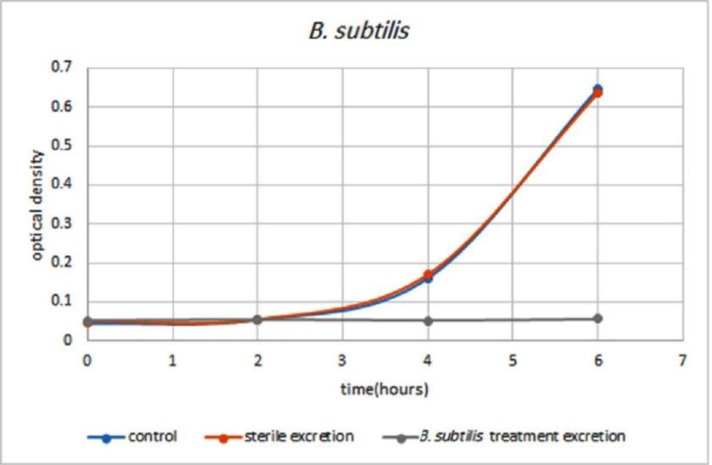
**Rate of bacterial growth.** Blue chart: growth of *B. subtilis* bacteria in vicinity of secretions of *B. subtilis* bacteria-treated larvae. Red chart: growth of *B. subtilis* bacteria in vicinity of secretions of sterile larvae. Gray graph: growth of *B. subtilis* as control.

Comparison and examination of the graphs of larval secretions-treated strains implicated that the *B. subtilis* treated larvae secretions was able to inhibit the growth of *B. subtilis* for 5 hours (Fig. 1) as well as *E. coli* treated larvae secretions was able to inhibit the growth of *E. coli* for less than 4 hours (Fig. 2). The secretions of Larval which grown in a sterile environment was also completely unsuccessful in inhibiting the growth of two strains of bacteria. The results of inhibition of *E. coli* and *B. subtilis* by larval secretions have been presented in Figs 1 and 2.

### Determination the role of pH in inhibiting bacterial growth.

pH of *B. subtilis* and *E. coli* bacteria-treated larvae secretions were measured. pH of *B. subtilis* treated larvae secretions was 8.99 as well as pH of *E. coli*-treated larvae secretion was 8.35. In this way, the alkaline nature of the larva secretions was confirmed in the study. Then, pH of mixture of 1ml Laura Bertani liquid culture medium with 1 ml *E. coli* bacteria-treated larvae secretions as well as pH of mixture of 1 ml Laura Bertani liquid culture medium with 1 ml *B. subtilis* bacteria-treated larvae secretions was measured. pH of mixture of 1 ml Laura Bertani liquid culture medium with 1 ml *E. coli*-treated larvae secretions was 7.26 and pH of mixture of 1 ml Laura Bertani liquid culture medium with 1 ml *B. subtilis*-treated larvae secretion was 7.77 as well. The results of the growth rate of *E. coli* and *B. subtilis* strains showed that after three hours growth of bacteria in an altered pH culture medium was inhibited by 10% to control samples. Whereas, the mixture of larvae secretions with LB culture medium were able to completely inhibit growth of bacteria for three hours.

### Investigation of gene expression.

**Fig. 3.**
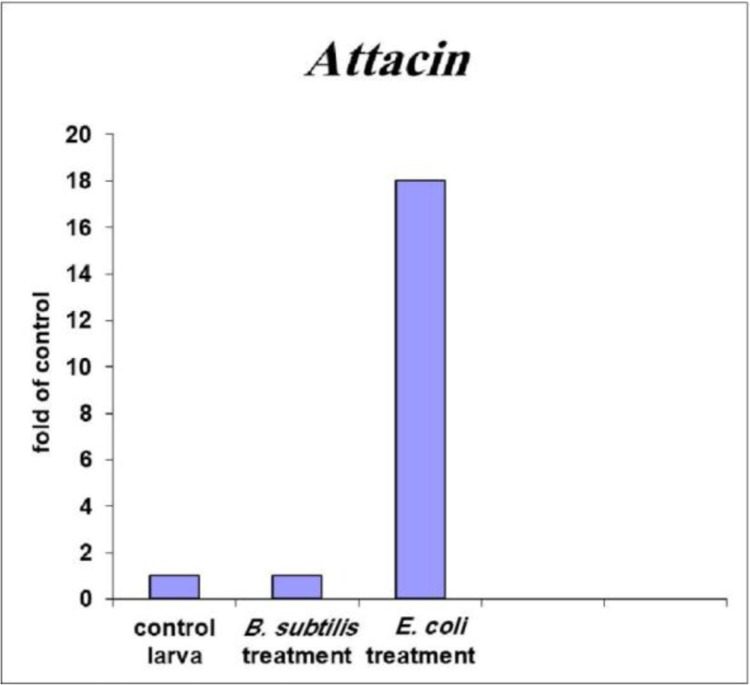
**Gene expression.** The difference in the expression of attacin gene in sterilized, *B. subtilis* and *E. coli*-treated larvae

**Fig. 4.**
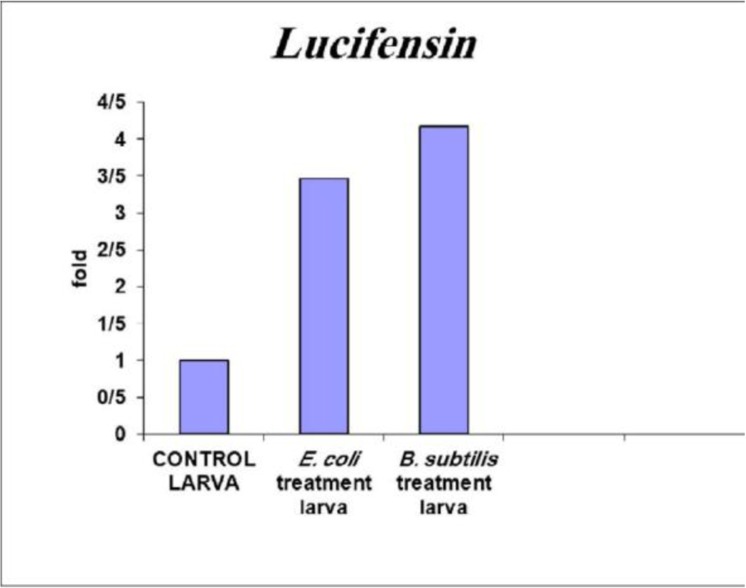
**Gene expression.** The difference in expression of the lucifensin gene in bacterial-treated and sterile larvae

After obtaining data related to the efficiency of primer of each gene and completion of the real-time PCR, using the pFaffelf method, expression of each gene relative to the reference gene was studied in control and bacteria-treated samples. The results indicated that the rate of the attacin gene expression in response to the *E. coli*-contaminated environment increased in comparison to the sterile and the *B. subtilis*-contaminated environments (Fig. 3). The expression of this gene in the *B. subtilis*-infected environment does not differ from the sterile environment. Expression of the lucifensin gene in response to the *B. subtilis* and *E. coli*-contaminated environment increased in comparison to the sterile environment (Fig. 4). Expression of this gene in the *B. subtilis*-contaminated environment was increased in comparison to the *E. coli*-contaminated environment.

## DISCUSSION

In the present study, placement of larvae in bacterial contamination was investigated in an area similar to the wound and the effect of such conditions on the production and secretion of antibacterial compounds was also investigated. This study showed that the entrance of bacteria into the gastrointestinal tract of larvae would stimulate the production of antibacterial compounds. These compounds are secreted out of the body of the larvae and eliminate bacterial contamination. Chronic wounds are often subject to high risk of contamination by clones from the natural flora of the body; however, contamination to antibiotic-resistant bacteria, such as *E. coli*, also leads to the increase in number of wound infections. Infection of wound to hospital pathogens requires topical and systemic treatments using antimicrobial drugs. But these drugs have a risk of side effects for the patient and it is the cause of infection of the wound by other resistant bacteria and even as a complicated problem can slow down the healing process and even can lead to patient death (12). The power of the *Lucilia sericata* larvae in eliminating infection and accelerating the recovery of chronic wound has been proven in clinical trials (13).

The present study showed that secretions of larvae grown in an *E. coli*-contaminated environment could inhibit the growth of *E. coli* strains for about 3 hours and secretions of larva grown in a *B. subtilis*-contaminated environment inhibited the growth of *B. subtilis* bacteria for about 5 hours. Afterwards, the inhibitory effect of these secretions is gradually reduced. These results confirm that the presence of antibacterial compounds in larval secretions have the ability to inhibit the growth of bacteria. Due to the fact that the large part of the antibacterial compounds of the larvae is produced in the salivary glands and is transported out of the body along with saliva as well as during obtaining the larval secretions and the antibacterial compounds remained completely intact and healthy, therefore, the success of larval secretions in inhibiting the growth of *E. coli* and *B. subtilis* bacterial strains can be understandable.

The reason that could explain the short duration of inhibitory effect of secretions on bacterial growth is that for obtaining digestive secretions, larvae were placed in distilled water for 12 hours and digestive secretions which entered into water were collected as well. By doing this, the larvae exit from the simulated wound area as well as antibacterial compounds of larval secretions enter into the distilled water and are diluted. If larvae are constantly present in the wound area during larval treatment, they will secret their new digestive secretions into the wound area with greater concentration (new antibacterial compounds will be replaced rather than the previous antibacterial compounds). In this study, due to the low concentration of effective compounds or their short half-life, as well as lack of replacement, the effects are temporary and they are measured over a short period of time. Also, due to the temperature range of 25–28°C for the life and growth of larvae, the optimal temperature for the activity of larval antibacterial compounds should probably be the same.

If the bacteria grow at 37°C, then high temperatures can reduce the activity of antibacterial compounds. Also, according to the results obtained in this study, larval secretions have alkaline pH. The mixture of larval secretions with bacterial culture medium, which should have a pH of 7, causes alteration in the final mixture of the culture medium and larvae secretions which undoubtedly affects the optimal activity of antibacterial compounds in larvae secretions and reduces their optimal activity.

In the past, the effects of extracts or secretions of sterile larvae in inhibiting bacterial growth have been investigated and in limited cases, the effect of extract or secretions of bacteria-treated larvae on bacterial growth has been investigated. Kerridge et al. showed that after placement of bacteria in the vicinity of sterile larval secretions, growth of antibiotic susceptible *Staphylococcus aureus* and A, B streptococcus was well-inhibited the Growth of antibiotic resistant *S. aureus* and *Pseudomonas aeruginosa* strains were slightly inhibited. The larvae secretions did not have any effect on the growth of *Enterococcus* sp while growth of *E. coli* was increased in the vicinity of larval secretions (14). It has been shown that the effect of sterile larval secretions at 4 concentrations (1, 2, 3 and 4 g/ml) on growth of *S. aureus, E. coli* and *P. aeruginosa* only, with concentration of 1 g/ml, did not have any effect on the growth of *S. aureus*, and other concentrations of larval secretions resulted in inhibiting growth of bacteria (15).

In another study, *Lucilia sericata* larvae were injured and contaminated by *E. coli*-derived lipopolysaccharide-impregnated Needle. The complete extract of the body of the sterilized and infected larvae was capable to inhibit the growth of the *M. luteus.* Bacteria-contaminated larvae extract was more powerful than extract of sterile larvae to inhibit the growth of *M. luteus* (16). Ciprofloxacin inhibited the growth of *S. aureus* at a concentration of 1 μg/ml (Which was equal to 100%). Sterile larval secretions also had an inhibitory effect on the growth of this bacterium. The inhibitory effect of larval secretions was increased at 60% and 80% concentration in combination with antibiotics. After six days, antibiotics with a concentration of 100% lost its inhibitory effect on bacterial growth, but larval secretions retained its inhibitory effect in inhibiting growth of bacteria (17).

The results obtained in this study are significantly different from results of previous researches. In the previous studies, the complete extract of the body of sterile larval, whose immune system was not stimulated to produce antibacterial compounds, could inhibit the growth of bacteria. The complete extract of the infected larvae was more effective than the sterile larvae in inhibiting the growth of bacteria. One reason for the difference in the results between this research and previous researches is that in previous studies, used antibiotic-susceptible bacteria or Bacteria were resistant only to antibiotics. Antibiotic-resistant bacteria are susceptible to certain antibacterial compounds and other antibacterial compounds have no effect on them. The bacteria used in this study were resistant to several antibiotics and in other words, it could be said that they were resistant to several functional mechanisms of antibacterial compounds.

Comparison of the function of sterile larvae and bacterial-infected secretions on inhibition of bacteria growth indicates that antibacterial compounds of larvae have inductive nature and the growth of larvae in a bacterial-contaminated environment leads to the production of antibacterial compounds in the larvae's body. The obtained results of the Bradford test indicated a low concentration of larval secretions, but these larval secretions had the ability to inhibit the growth of antibiotic-resistant bacteria. This indicates that the antibacterial compounds present in the larval secretions are very powerful, which can be replaced rather than existing disinfectants drugs.

Another investigated issue in previous studies is the difference between the effect of larvae extracts and larvae secretions in inhibiting growth of Gram-positive and Gram-negative bacteria. The results of previous experiments show that larvae or their extracts are more powerful to inhibit the growth of Gram-positive bacteria in comparison to Gram-negative bacteria (18, 19). In this study, larvae secretions had somewhat more success in inhibiting the growth of Gram-positive *B. subtilis* than inhibition of growth of Gram-negative *E. coli*. Larvae secretions inhibited the growth of *B. subtilis* bacteria for 5 hours and they inhibited the growth of *E. coli* for less than 4 hours as well. Endotoxins produced by Gram-negative bacteria can destroy antibacterial compounds in larval secretions and also the thickness of the wall structure of these bacteria can be a cause of higher resistance of Gram-negative bacteria to larval secretions (18).

Larvae inhibit the growth of bacteria by alkalizing the environment and producing antibacterial compounds. By determining the effect of the changed pH-culture medium on inhibition of the bacterial strains growth, we can determine the effect of the alkaline pH of the larval secretions in inhibiting bacterial growth. In other words, it is possible to determine what proportion of the inhibitory effects belongs to the alkaline pH and what proportion is related to antibacterial compounds present in larval secretions. In this study, it was stated that the rate of bacterial growth in the mixture of culture medium with larvae secretions is completely inhibited for 4 hours (on average).

The growth of bacteria was reduced in a culture medium which its pH was elevated and was more alkaline in comparison to bacteria that were in normal culture medium with constant pH but this growth of bacteria was not completely inhibited. These results indicate that the compounds which are responsible for creating the alkaline environment of larval secretions, in addition to their task to creating alkaline environment, they have also another activity in inhibiting bacterial growth. The lack of such altered factors in the altered pH culture medium can explain reducing the inhibitory effect of larval secretions.

In previous studies, lucifensin and attacin compounds have also been studied. The level of the attacin gene expression in the larvae which was injured by *P. aeruginosa*-contaminated needles increased in comparison to the larvae which had not been injured by needles. The level of the defensin-1 gene expression (a member of the Defensin family) in the larvae which was injured by *P. aeruginosa*-contaminated needle also increased compared to the larvae which had not been injured. The increase in expression of this gene was due to the larvae's response to needle-created injuries not to the contamination of Gram-negative bacteria (20).

According to the results obtained in the present study, the expression level of the lucifensin gene (a member of the defensin family) has also increased significantly in the Gram-negative bacteria-infected environment, it can be said that in the experiment conducted by Baumann et al. (20), the expression level ofdefensin-1 gene increased in response to the Gram-negative bacterium-created infection. Therefore, it can be concluded that, contrary to the limitations and low strength of the antibacterial compounds of the defensin family in inhibiting the growth of Gram-positive bacteria, some members of dysfunctions family, including lucifensin, are also active against Gram-negative bacteria (20).

In this research, the rate of attacin gene expression showed more changes in response to the bacterial infection than the lucifensin gene. The reason that can explain this difference is that the collective function of some antibacterial compounds of insects is more than the function of each of these compounds alone. This incremental effect results in reduction of the needed amount of each compound to fully cope with bacterial infection as well as reduction of the cost of the immune system (13). The bacteria used in this study were multi drug-resistant bacteria and may have been resistant to several antibacterial compounds in the larvae which have functions similar to these antibiotics as well as the larval body has greatly increased the level of attacin expression to compensate this deficiency. Therefore, attacin is responsible for this task.

## CONCLUSION

In general, larval therapy is a powerful method for the removal of wound infections and chronic wound healing. Clinical experience suggests that larval therapy reduces the patient's need for antibiotic or reduces duration of hospitalization after surgery (22). However, the use of living creatures in this method has led to some disadvantages including pain. Additionally, larvae cannot be used to heal all wounds like bleeding wounds (13). There are some benefits of replacing larva secretions rather than live larvae include:

A more predictable and more uniform product is available and it's easier to use and perhaps is more cost-effective (Extract and secretions of larvae can be maintained for a long time). In addition, due to the lack of use of live larvae, the patient will feel better in this type of treatment. If extracts are used instead of live larvae, larvae of other species can be used and by replacing the extract rather than live larvae as well as by maintaining the beneficial effects of other larvae, the risk of attacking healthy tissue is eliminated.

Due to the fact that the precise function of the extract and secretions of the larva is not clearly understood, the exact identification of the function mechanism and the type of reaction of the extract and secretions of the larvae to the factors present in the chronic wound is important. In summary, this study confirmed the effect of larvae secretions in eliminating infection and on inhibition of growth of several antibiotic resistant bacteria and it can be certainly said that the presence of the larvae in the bacteria-infected environment can cause increased expression of the antibacterial compounds. Therefore, larval secretions have the potential to become a drug for treatment of several antibiotic-resistant bacteria and this power and potential are increased by placement of larvae in an infected environment.
